# An exploratory study of the management strategies reported by endurance athletes with exercise-associated gastrointestinal symptoms

**DOI:** 10.3389/fnut.2022.1003445

**Published:** 2022-11-09

**Authors:** Rachel Scrivin, Ricardo J. S. Costa, Fiona Pelly, Dana Lis, Gary Slater

**Affiliations:** ^1^School of Health and Behavioral Sciences, University of the Sunshine Coast, Sippy Downs, QLD, Australia; ^2^Faculty of Health, Education and Environment, Toi Ohomai Institute of Technology, Tauranga, New Zealand; ^3^Department of Nutrition Dietetics and Food, Monash University, Melbourne, VIC, Australia; ^4^Department of Neurobiology, Physiology and Behavior, University of California, Los Angeles, Los Angeles, CA, United States

**Keywords:** athletes, dietary strategies, dietary fiber, exercise, gastrointestinal symptoms, questionnaire, Ex-GIS, low FODMAP

## Abstract

This exploratory study investigated endurance athletes self-reported exercise-associated gastrointestinal symptoms (Ex-GIS) and associated strategies to manage symptomology. Adult endurance athletes with a history of Ex-GIS (*n* = 137) participating in events ≥ 60 min completed an online validated questionnaire. Respondents included runners (55%, *n* = 75), triathletes (22%, *n* = 30), and non-running sports (23%, *n* = 32), participating at a recreationally competitive (37%, *n* = 51), recreationally non-competitive (32%, *n* = 44), and competitive regional/national/international (31%, *n* = 42) levels. Athletes identified when Ex-GIS developed most frequently either around training (AT), around competitions (AC), or equally around both training (ET) and competitions (EC). Athletes reported the severity of each symptom before, during, and after exercise. Athletes predominantly categorized Ex-GIS severity as mild (< 5/10) on a 0 (no symptoms) to 10 (extremely severe symptoms) visual analog symptomology scale. The Friedman test and *post hoc* analysis with Wilcoxon signed rank test was conducted with a Bonferroni correction applied to determine differences between repeated measures. The only severe symptom of significance was the urge to defecate during training in the ET group (*Z* = –0.536, *p* = 0.01). Ex-GIS incidence was significantly higher during training and competitions in all categories. A content review of self-reported strategies (*n* = 277) to reduce Ex-GIS indicated popular dietary strategies were dietary fiber reduction (15.2%, *n* = 42), dairy avoidance (5.8%, *n* = 16), and a low fermentable oligosaccharides, monosaccharides, and polyols (FODMAP) diet (5.4%, *n* = 15). In contrast, non-dietary strategies included the use of medications (4.7%, *n* = 13) and relaxation/meditation (4.0%, *n* = 11). On a Likert scale of 1–5, the most successful dietary strategies implemented were dietary fiber reduction (median = 4, IQR = 4, 5), low FODMAP diets (median = 4, IQR = 4, 5), dairy-free diets (median = 4, IQR = 4, 5), and increasing carbohydrates (median = 4, IQR = 3, 4). Accredited practicing dietitians were rated as the most important sources of information for Ex-GIS management (*n* = 29). Endurance athletes use a variety of strategies to manage their Ex-GIS, with dietary manipulation being the most common.

## Introduction

Participation in endurance and ultra-endurance sport has increased steadily over the last few decades (1). With the increase in event participation, there has been a corresponding increase in the reported incidence of performance-debilitating exercise-associated gastrointestinal symptoms (Ex-GIS), proportional to the physiological demands of the exercise stress (2–5). The pathophysiological mechanisms for Ex-GIS originate from a dynamic and multifaceted gastrointestinal and systemic perturbations network previously described within the exercise-induced gastrointestinal syndrome model (2, 5, 6). These gastrointestinal disturbances occur via the neuroendocrine and circulatory-gastrointestinal pathways or through mechanical strain on the peritoneal cavity due to exercise (5, 6).

Self-reported Ex-GIS during or after exercise can vary from mild inconvenience to severe. When severe, Ex-GIS may compromise fueling and hydration strategies during exercise (7), impair athletic performance, or lead to the cessation of exercise (8), eventuating in event withdrawal (8, 9). The clinical and performance significance of Ex-GIS have been previously documented (5, 7, 10, 11). For example, Ex-GIS may indicate gastrointestinal integrity or functional issues that warrant medical attention (12). In addition, Ex-GIS incidence and severity have been shown to impair exercise performance in controlled laboratory experimental models (7, 8, 12).

Ex-GIS can typically present in the upper or lower gastrointestinal tract regions. It can also present as other related symptoms such as nausea, dizziness, and acute transient abdominal pain (5, 13). The incidence of Ex-GIS reported by endurance and ultra-endurance athletes within specific populations ranges from 4 to 96% of athletes and is dependent on intrinsic and extrinsic factors (2, 5, 6, 9, 14). Intrinsic factors that may increase the incidence and extent of Ex-GIS include biological sex (i.e., females exercising in the luteal or early menstrual phase of the menstrual cycle) (15), hypohydration (16, 17), individual feeding tolerance (7, 8, 11), predisposing or active functional gastrointestinal disease or disorders (5), gastrointestinal bacterial composition (e.g., the gut microbiome) (18), and dietary intake around exercise (7, 9). Extrinsic factors that may exacerbate symptoms include the type of exercise (i.e., higher reported incidence in runners) (14), longer duration and/or greater exercise stress (9), hot temperatures (2, 19, 20), nocturnal exercise (21), and non-steroidal anti-inflammatory drug use (2, 22).

Several specific dietary factors may influence the development of Ex-GIS around exercise, including individual food components (10, 23) and macronutrient types (24). The influence of these dietary factors depends on an individual’s gastrointestinal tolerance, amounts consumed, and the timing of consumption. Endurance athletes have been known to manipulate specific food components in their diets to reduce Ex-GIS, such as gluten-free (25, 26) or low fermentable oligosaccharide disaccharide monosaccharide and polyols (FODMAP) diets (25, 27, 28). Laboratory studies have shown that when endurance athletes consume a short-term gluten-free diet, there was no difference in performance, gastrointestinal symptoms, or intestinal injury compared to the gluten-containing diet (23). In contrast, preliminary research indicates a low FODMAP diet 24 h before endurance exercise in the heat results in less Ex-GIS and malabsorption compared to a high FODMAP diet. However, a novel finding of that study is that the high FODMAP diet ameliorated intestinal epithelial injury (10). Further research is required in this area to determine the significance of this finding.

Other dietary factors which may reduce Ex-GIS are the types and amounts of macronutrients consumed during endurance exercise. Research has shown that when 15 g of carbohydrate or whey protein hydrolysate is consumed pre-exercise and every 20 min while running (i.e., 45 g/h) at 60% of VO_2max,_ both macronutrients ameliorate intestinal injury and permeability, improve endotoxin clearance, and reduce stress markers compared to water (24). However, endurance athletes reported fewer Ex-GIS in the carbohydrate group, which suggests protein is less well tolerated during endurance exercise (5, 24). Therefore, it appears that moderate amounts of carbohydrates are (10) well tolerated during endurance exercise; however, higher carbohydrate intake rates (up to 90 g/h as advocated during endurance exercise) (29–31) are often less well tolerated (11), unless countermeasures such as gut training are implemented (7, 8). In addition to dietary factors, maintaining euhydration before (32) and during endurance exercise has been shown to reduce Ex-GIS and improve gastrointestinal integrity and function compared to dehydrated controls (16). Athletes may also seek non-dietary factors (e.g., relaxation or meditation) to manage Ex-GIS, which requires further investigation.

Many factors may influence Ex-GIS development; therefore, athletes may often seek different ways of managing the incidence and severity of Ex-GIS. Due to their nutrition expertise, it is possible that accredited practicing dietitians are a preferred source of nutrition information for athletes. However, this likely depends on accessibility and background, i.e., country of origin (33–35). Interestingly, social media, team-mates, or other athletes also appear to be popular information sources for athletes (35–37), possibly due to the convenience and ease of access to these sources. Less is known about the preferred information sources for athletes who report Ex-GIS, as it often involves dietary and non-dietary management (5, 12).

The primary aim of the current research was to investigate self-selected dietary and non-dietary strategies endurance athletes use to manage Ex-GIS and their preferred source of information about these strategies.

## Materials and methods

### Experimental design

This exploratory study used a previously established validated online questionnaire to investigate self-reported Ex-GIS, management strategies, and information sources (38). Inclusion criteria were endurance athletes ≥ 18 years of age, training and competing at any level in events ≥ 60 min, who experience Ex-GIS. Athletes were recruited internationally through professional and academic networks. Endurance athletes accessed the questionnaire online through the Qualtrics Survey Platform (RRID:SCR_016728) (Qualtrics LLC, 333 West River Park Drive Provo, UT 84604, United States). All responses collected were anonymous. This study was approved by the Human Research Ethics Committee (University of the Sunshine Coast, Australia), ethics approval number S201402 and conformed to the Helsinki Declaration for Human Research Ethics.

The questionnaire included participant information (e.g., biological sex, age, main endurance sport, participation level, and event characteristics, i.e., single-day or multi-day), Ex-GIS (including the onset of symptoms, symptom incidence and severity, and information sources for Ex-GIS) (1–21 items), intended practices for Ex-GIS (9 items), and diseases or disorders of the gastrointestinal tract (3 items). The questionnaire included 43 items depending on dichotomous, Likert scales, or descriptive responses (38) (Supplementary material 1).

Retrospectively, athletes selected when they experienced Ex-GIS the most frequently, either around training (AT) or around competitions (AC) or equally around both training and competitions. Athletes indicating Ex-GIS equally around both training and competitions completed both training and competition-specific questions, which were categorized as equally training (ET) and equally competition (EC). Athletes then responded to questions on when Ex-GIS occurred (i.e., before, during, or after exercise) and the severity of the Ex-GIS.

A validated modified visual analog scale symptomology assessment tool was used for determining the incidence and severity of Ex-GIS in response to exercise stress (13). The symptom severity scale was from 0 to 10; 0 no symptoms, 1–4 mild symptoms (i.e., the sensation of Ex-GIS but not substantial enough to interfere with exercise workload), 5–9 severe symptoms (i.e., Ex-GIS substantial enough to interfere with exercise workload) to 10 extremely severe symptoms (i.e., indicative of extreme Ex-GIS warranting exercise cessation). Athletes selected the severity of each symptom (from 0 to 10) whereby the incidence was.

Athletes completed open-field response questions on dietary and non-dietary strategies tried to reduce Ex-GIS. A content review process was applied for all qualitative data, categorizing and coding responses into the most common dietary and non-dietary themes (39). The themes were the five most common dietary strategies to reduce Ex-GIS around exercise, specific high FODMAP food group exclusion, and the non-dietary strategies used to manage Ex-GIS. From pre-defined lists, athletes selected the most successful dietary components (e.g., macronutrients), dietary strategies (e.g., dairy-free diets), and supplements (e.g., probiotics) and when they consumed these (e.g., before, during, and after exercise).

### Statistical analysis

The type of sport and event characteristics data were collapsed when categories had more than 20% of the cells with less than an expected count of five. For example, cycling, swimming, walking, multisport, and other sports were collapsed into a new category, “non-running sports,” and competitive regional, national, and international participation groups collapsed into a new category CompRNI.

Athletes who experienced Ex-GIS equally in training and competitions completed both training and competition Ex-GIS severity questions, i.e., repeated measures in this category. Therefore, four groups were used for data analysis, i.e., AT, AC, ET, and EC. Repeated measures were also applied within each symptom category, i.e., symptom incidence and severity before, during, and after training or competition. The incidence was calculated as the number of athletes responding to each symptom in each group, and the severity was calculated as the average response to each symptom.

Descriptive statistics were used to define continuous variables (means, standard deviations, and percentages), and for non-parametric data, medians and interquartile ranges (IQR) were applied. Significance was accepted with α ≤ 0.05. Chi-squared tests were used to determine associations between categorical data, i.e., main sport, participation level, event characteristics, and biological sex. For any significant group differences, Bonferroni *post hoc* tests were used. For Ex-GIS incidence and severity, the data were checked for normality by applying the Shapiro–Wilk test. The Friedman test was used to determine differences within each symptom group, and a *post hoc* Wilcoxon test to determine differences within repeated measures. A Bonferroni correction factor was applied if significant. Data analysis was performed using IBM SPSS Statistics 27.0 (RRID:SCR_019096) (IBM Corporation New Orchard Road Armonk, NY 10504-1722, United States).

## Results

One hundred and thirty-seven endurance athletes with Ex-GIS completed the questionnaire. More females (63%, *n* = 86) completed the questionnaire than males (37%, *n* = 51) (*p* < 0.001). The mean age was 41.6 ± 11.1 years, and there was no difference in mean age between females (42.1 ± 11.1 years) and males (40.6 ± 11.1 years) (*p* = 0.58). Participant characteristics are described in Table 1, and Ex-GIS incidence in Table 2. There were no differences in Ex-GIS incidence by main sport, participation level, or biological sex.

**TABLE 1:** Participant characteristics of endurance athletes with exercise-associated gastrointestinal symptoms (*n* = 137 respondents).

		Main sport			Participation level			Event type		Sex	
	Totals *n*	Running *n*, (%)	Triathlon *n*, (%)	Non-running sports *n*, (%)	Rec. Non-comp *n*, (%)	Rec. Comp *n*, (%)	Comp RNI *n*, (%)	Single day	Multi-day ≥ 2 days	M *n*, (%)	F *n*, (%)
Demographics	137	75 (55)	30 (22)	32 (23)	44 (32)	51 (37)	42 (31)	129 (94)	8 (6)	51 (37)	86* (63)

*Significant difference; Comp, Competitive; CompRNI, Competitive regional, national and international; *n*, number; Rec., recreational; %, percentage; ≥, greater than or equal to.

**TABLE 2:** Incidence of exercise-associated gastrointestinal symptoms development for the main sport, participation level, and gender (*n* = 137 respondents).

		Main sport				Participation level				Sex		
Incidence of Ex-GIS development	Totals *n*	Running *n*, (%)	Triathlon *n*, (%)	Non-running sports *n*, (%)	Assoc. between Ex-GIS develop. and main sport χ^2^ (*p*)	Rec. non-comp *n*, (%)	Rec. comp *n*, (%)	CompRNI *n*, (%)	Assoc. between Ex-GIS develop. and part. level χ^2^ (*p*)	Male *n*, (%)	Female *n*, (%)	Assoc. between Ex-GIS develop. and gender χ^2^ (*p*)
Around training	29 (21)	17 (58)	4 (14)	8 (28)	1.19 (0.55)	8 (28)	12 (41)	9 (31)	0.32 (0.85)	10 (35)	19 (65)	0.09 (0.76)
Around competitions	41 (30)	18 (44)	14 (34)	9 (22)	3.68 (0.16)	10 (24)	10 (24)	21 (52)	8.21 (0.02)*	20 (49)	21 (51)	2.3 (0.13)
Equally around training and competitions	67 (49)	40 (60)	12 (18)	15 (22)	0.82 (0.67)	26 (39)	29 (43)	12 (18)	5.14 (0.08)	21 (31)	46 (69)	0.97 (0.32)

*No significant difference after *post hoc* Bonferroni correction (*p* < 0.05). Assoc., association; Comp, Competitive; CompRNI, Competitive regional, national, and international; Develop., development; Ex-GIS, exercise-associated gastrointestinal symptoms; *n*, number; part., participation; *p*, probability value; Rec., recreational; %, percentage; χ^2^, chi-square statistic.

**FIGURE 1:**
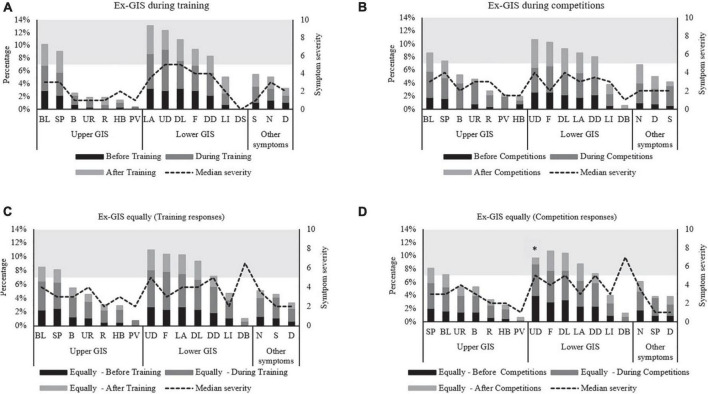
Combined incidence and median severity rating of gastrointestinal symptoms before, during, and after exercise. Symptoms are listed in upper, lower, and other symptom categories and descending order of incidence. Symptom severity scale is from 0 to 10; 0 = no symptoms, 1–4 mild symptoms, (i.e., the sensation of Ex-GIS but not substantial enough to interfere with exercise workload), 5–9 severe symptoms, (i.e., Ex-GIS substantial enough to interfere with exercise workload/grayscale and above) to 10 extremely severe symptoms, (i.e., indicative of extreme Ex-GIS warranting exercise cessation). **(A)** Ex-GIS around training only, **(B)** Ex-GIS around competitions only, **(C)** Ex-GIS equally around training and competitions (training responses). **(D)** Ex-GIS equally around training and competitions (competition responses). *Significant difference before and during competitions for UD. B, Belching; BL, Bloating; D, Dizziness; DB, Defecation Bloody Stools; DD, Defecation (Diarrhea); DL, Defecation Loose Stools; F, Flatulence; Ex-GIS, exercise-associated gastrointestinal symptoms; HB, Heartburn; LA, Lower Abdominal Bloating; LI, Left Intestinal Pain; N, Nausea; PV, Projectile Vomiting; R, Regurgitate; S, Stitch; SP, Stomach Pain; UD, Urge to Defecate; UR, Urge to Regurgitate.

Figure 1 highlights the combined incidence for each Ex-GIS and the median severity of all symptoms across all independent groups. Lower-GIS was more commonly reported compared to upper-GIS in all groups, i.e., AT (*p* = 0.006), around competition (*p* = 0.04) and EQ AT (*p* = 0.02), and EQ competition (*p* = 0.01).

The incidence and severity of symptoms were not normally distributed; therefore, median and IQR were used to describe the data with non-parametric tests applied. Using the Wilcoxon test for non-parametric data the incidence of reported symptoms was significantly greater during exercise than before and after exercise (e.g., AT, AC, ET, EC) (Table 3)., The urge to defecate during competitions in the EC group was the only severe symptom of significance after *post hoc* testing (*Z* = –0.536, *p* = 0.01). Overall, the median severity rating for most Ex-GIS was mild (median < 5/10) (Figure 1).

**TABLE 3:** Exercise-associated gastrointestinal symptoms incidence reported by endurance athletes before, during and after training or competition (*n* = 137 respondents).

Incidence of Ex-GIS	Timing	Ranks	Ranks (*n*)	Mean rank	Sum of ranks	Z score	Effect size	Significance (*p*)
Around training only	DT—BT	Negative	1	14	14	–2.62*	–0.45	0.009
		Positive	14	7.57	106			
		Ties	2					
	AT—BT	Negative	3	5.67	17	–1.10*	–0.19	0.27
		Positive	7	5.43	38			
		Ties	7					
	AT-DT	Negative	12	7.67	92	–2.50^†^	–0.43	0.012
		Positive	2	6.5	13			
		Ties	3					
Around competitions only	DC—BC	Negative	1	1.5	1.5	–3.55*	–0.61	<0.001
		Positive	16	9.47	151.5			
		Ties	0					
	AC—BC	Negative	2	3.25	6.5	–3.24*	–0.55	0.001
		Positive	14	9.25	129.5			
		Ties	1					
	AC—DC	Negative	12	9.63	115.5	–2.46^†^	–0.42	0.014
		Positive	4	5.13	20.5			
		Ties	1					
Equally around training and competitions—training responses only	DT—BT	Negative	0	0	0	–3.64*	–0.62	<0.001
		Positive	17	9	153			
		Ties	0					
	AT—BT	Negative	9	8.94	80.5	–1.18^†^	–0.20	0.24
		Positive	6	6.58	39.5			
		Ties	2					
	AT—DT	Negative	17	9	153	–3.63^†^	–0.62	<0.001
		Positive	0	0	0			
		Ties	0					
Equally around training and competitions—competition responses only	DC—BC	Negative	0	0	0	–3.53*	–0.61	<0.001
		Positive	16	8.5	136			
		Ties	1					
	AC—BC	Negative	7	10.21	71.5	–0.19^†^	–0.03	0.85
		Positive	9	7.17	64.5			
		Ties	1					
	AC—DC	Negative	15	8	120	–3.41^†^	–0.59	<0.001
		Positive	0	0	0			
		Ties	2					

*Based on negative ranks.

^†^Based on positive ranks. AC, around competition; AT, around training; BC, before competition; BT, before training; DC, during competition; DT, during training; *n*, number; *p*, probability value; *Z*, z statistic; <, less than.

**FIGURE 2:**
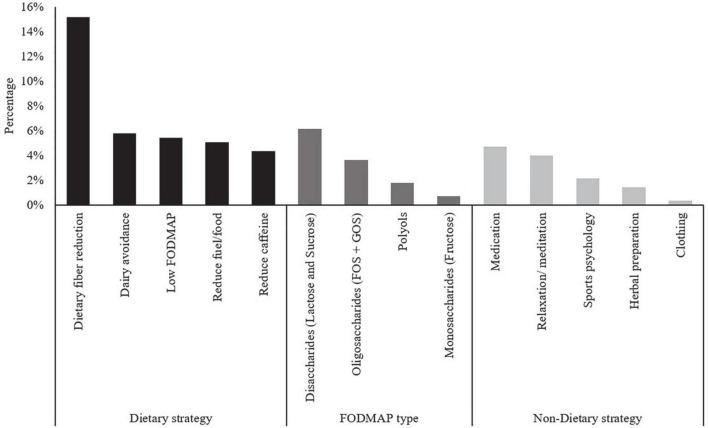
Content review of endurance athletes’ five most common dietary strategies, including specific FODMAP groups described, and non-dietary strategies used to reduce exercise-associated gastrointestinal symptoms. FODMAP, fermentable oligosaccharide disaccharide monosaccharide and polyols; GOS, galacto-oligosaccharides; FOS, fructo-oligosaccharide.

Sixty-seven percent (*n* = 92) of athletes reported using dietary or non-dietary strategies to manage their Ex-GIS. A total of 277 strategies were reported by athletes, an average of 3.0 ± 1.6 strategies per athlete. Of those who reported using dietary or non-dietary methods, 77% (*n* = 71) of athletes self-reported using only dietary strategies to reduce Ex-GIS (often multiple dietary strategies listed). A combination of both dietary and non-dietary strategies was reported by 17% (*n* = 16), while 5% (*n* = 5) of athletes reported only trialing non-dietary methods. A qualitative content review of the strategies used including the five most common dietary strategies to reduce Ex-GIS, specific high FODMAP food groups exclusion, and the non-dietary strategies used to reduce Ex-GIS, are shown in Figure 2. The most popular dietary strategies were dietary fiber reduction (15.2%, *n* = 42) and dairy avoidance (5.8%, *n* = 16), whereas non-dietary strategies included the use of medications (4.7%, *n* = 13) and relaxation/meditation (4.0%, *n* = 11). Avoiding disaccharides (lactose and sucrose) (6.1%, *n* = 17) were the most popular FODMAPs to avoid or reduce.

Endurance athletes rated the overall success of specific dietary components (Table 4) or attempted dietary strategies (Table 5) and when they tended to implement them. Significantly less dietary fiber (*p* = 0.05) and fat (*p* = 0.04) was consumed before, during, and after exercise, and significantly more water (*p* = 0.01) was consumed (at all-time points) to reduce Ex-GIS.

**TABLE 4:** Dietary components self-selected to eat more or less of to reduce the development of exercise-associated gastrointestinal symptoms.

Dietary component	The success of dietary components in reducing Ex-GIS Median (IQR)* (*n*)	Before exercise (last meal or snack before exercise), *n*		During exercise, *n*		After exercise (within 30 min of exercise completion), *n*		More compared to less responses around exercise	Dietary change made but not specifically related to exercise, *n*		Not tried this dietary component, *n*
							
		More	Less	More	Less	More	Less	Significance (*p*)	More	Less	
Dietary fiber	4.0 (4.0, 5.0) (*n* = 48)	4	50	3	43	5	24	0.05	11	15	30
Carbohydrate	4.0 (3.0, 4.0) (*n* = 46)	37	15	33	24	25	6	0.06	16	10	31
Fat	4.0 (3.0, 4.0) (*n* = 35)	5	37	5	33	2	18	0.04	5	13	41
Water or fluid	4.0 (3.0, 4.0) (*n* = 40)	38	8	30	10	27	1	0.01	21	1	37
Coffee or caffeine	3.0 (3.0, 4.0) (*n* = 40)	10	38	9	29	2	20	0.43	3	21	39
Protein	3.0 (3.0, 4.0) (*n* = 31)	11	25	6	27	21	4	0.53	15	5	46

Likert Scale used to measure the success of dietary components (1, significantly worse; 2, somewhat worse; 3, no improvement; 4, some improvement; 5, significant improvement); CI, confidence interval; Ex-GIS, exercise-associated gastrointestinal symptoms; IQR, interquartile range; *n*, number; *p*, probability value.

**TABLE 5:** Specific dietary strategies trialed, and supplements used to reduce the development of exercise-associated gastrointestinal symptoms (listed in order of success at reducing symptoms).

	The success of dietary components in reducing Ex-GIS Median (IQR), (*n)*	Before exercise (last meal or snack before exercise), *n*	During exercise, *n*	After exercise (within 30 min of exercise completion), *n*	Diet or supplement tried, but not specifically related to exercise, *n*	Not tried this dietary strategy or supplement, *n*
**Dietary strategies**						
Dairy-free	4.0 (4.0, 5.0) (*n* = 30)	22	16	11	23	53
Low FODMAP	4.0 (4.0, 5.0) (*n* = 20)	17	12	11	18	63
Lactose-free	4.0 (3.0, 5.0) (*n* = 24)	15	12	9	20	59
Gluten-free	4.0 (3.0, 4.0) (*n* = 22)	16	13	9	20	58
Wheat-free	3.5 (3.0, 4.0) (*n* = 16)	12	9	6	16	64
**Supplementation**						
Arginine	5.0 (5.0, 5.0) (*n* = 1)	1	1	2	0	76
Probiotics	4.0 (3.0, 4.0) (*n* = 26)	9	0	5	27	47
Prebiotics	4.0 (3.0, 4.0) (*n* = 7)	0	0	1	10	71
Antioxidant	3.5 (3.0, 4.0) (*n* = 10)	5	2	3	12	64
L-Citrulline	3.0 (3.0, 5.0) (*n* = 3)	2	2	3	0	76
Glutamine	3.0 (3.0, 4.5) (*n* = 5)	3	2	2	4	72
Curcumin	3.0 (3.0, 4.0) (*n* = 7)	2	1	3	11	68
Nitrates	3.0 (1.25, 3.0) (*n* = 12)	11	2	5	8	64
Synbiotics	0	0	0	0	0	77
Bovine colostrum	0	0	0	0	0	77

Likert scale used to measure success (1, significantly worse; 2, somewhat worse; 3, no improvement; 4, some improvement; and 5, significant improvement); CI, confidence interval; Ex-GIS, exercise-associated gastrointestinal symptoms; FODMAP, fermentable oligosaccharide disaccharide monosaccharide and polyols; IQR, interquartile range; *n*, number.

**FIGURE 3:**
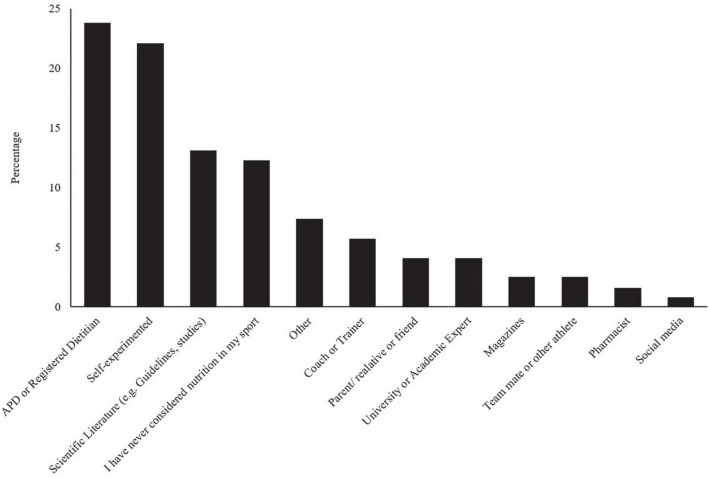
Endurance athletes’ most important information sources for managing exercise-associated gastrointestinal symptoms around exercise (*n* = 122 respondents). APD, Accredited Practicing Dietitian.

The most common sources of dietary information for Ex-GIS management are shown in Figure 3. After chi-squared analysis and *post hoc* testing, no significant associations were found between the most important nutrition information sources for managing Ex-GIS, categorized by main sports, participation levels, event characteristics, or biological sex.

Fifteen athletes (11%) responded that they had a diagnosed gastrointestinal disease or disorder. The most common medical or clinical condition was irritable bowel syndrome (IBS) (*n* = 6), comprising IBS-constipation predominant (*n* = 4) and IBS-diarrhea predominant (*n* = 2). Thirteen (9%) athletes from the entire cohort take medications (e.g., Omeprazole, Buscopan, Gaviscon) to manage their Ex-GIS, and six (4%) of those have a diagnosed gastrointestinal disease or disorder.

## Discussion

This is the first exploratory study to review the specific self-reported strategies used to manage symptomology amongst endurance athletes who experience Ex-GIS. The most commonly reported successful dietary strategies to manage Ex-GIS, typically before and during exercise, were dietary fiber reduction, a low FODMAP diet, a dairy-free diet, and increasing carbohydrates. Endurance athletes primarily sought accredited practicing dietitians in the management of Ex-GIS.

Reducing dietary fiber, particularly before exercise, was the most common dietary strategy endurance athletes have implemented. Current sports nutrition guidelines recommend endurance athletes reduce dietary fiber around key training sessions and competitions to mitigate the incidence of Ex-GIS (3, 29, 31). This is due to the ability of dietary fiber to increase the luminal contents in the large colon due to an osmotic effect and fermentation, which may promote greater gastrointestinal discomfort and reduced orocaecal transit times (5, 7). A low dietary fiber diet (<10 g/d) can reduce fecal matter and gastrointestinal secretions and thereby extend gastrointestinal tract transit time (40). However, due to variable gastrointestinal tract transit times, a low-fiber diet may require implementation 1–3 days before exercise (41), which may not be practical habitually. Furthermore, adult women and men are recommended to consume 25–30 g/d of dietary fiber to ensure proper gastrointestinal tract function and reduce the risk of several chronic diseases, including diabetes, heart disease, and certain cancers (42). Therefore, if athletes restrict dietary fiber to reduce Ex-GIS, a short-term low-fiber diet around specific competitions is likely to be practical without compromising the beneficial effects of consuming adequate dietary fiber daily.

Ex-GIS reported by athletes in this investigation was similar to individuals diagnosed with IBS, including abdominal pain, bloating, and diarrhea (43). An effective treatment strategy for those with IBS is implementing a low FODMAP diet (43, 44). Similarly, athletes commonly reported using a low FODMAP diet to manage Ex-GIS successfully in the current study. A low FODMAP diet has also reportedly been used to manage Ex-GIS in other investigations with athletes (25, 26). However, the time frame for implementation in previous studies, including the current study, has not been investigated. It has been shown that a short-term (24-h) low FODMAP diet before endurance exercise can reduce Ex-GIS and malabsorption (10), and the impact of this short-term dietary restriction on overall nutritional status is unlikely to be significant. However, a low FODMAP diet is not designed to be followed long-term due to possible nutritional deficiencies if chronically administered (43, 44). However, if a low FODMAP diet is necessary over more extended time frames (i.e., >24 h), integrating a rechallenge and personalization phase would identify which FODMAP foods require moderation, thereby providing less dietary restriction, e.g., dairy avoidance.

Dairy avoidance around exercise was an exclusive dietary strategy employed by athletes to reduce Ex-GIS, i.e., not related to the low FODMAP dietary cohort. In this study, dairy products were avoided before, during, and after exercise. Endurance athletes may have been avoiding dairy products due to the lactose component, which is typically moderated on a low FODMAP diet (43). It is possible that endurance athletes avoid dairy as they have lactose intolerance. Lactose intolerance is one of the most commonly reported food intolerances, with many symptoms overlapping with IBS, e.g., abdominal cramps, diarrhea, and bloating (45). In the current study, implementing a lactose-free diet was also reported as a successful dietary strategy to manage Ex-GIS; however, slightly lower implementation rates before, during, and after exercise were reported compared to a dairy-free diet. Future research could also investigate if athletes have food allergies or intolerances, hence the need for a dairy or lactose-free diet.

Many endurance athletes also reported successfully increasing carbohydrate around endurance exercise to mitigate Ex-GIS development. Increasing carbohydrate intake around exercise is a well-advocated dietary method to facilitate carbohydrate availability, particularly for endurance and ultra-endurance athletes (3, 29, 31). However, research has also shown that carbohydrate consumed during exercise facilitates splanchnic region blood flow (46). It is possible this also promotes intestinal epithelial blood flow, reducing epithelium damage. Indeed, carbohydrate or whey protein hydrolysate (WPH) ingestion before and during exercise ameliorates intestinal epithelial injury and reduces small intestine permeability (24), which may also indicate maintenance of splanchnic blood flow. However, given Ex-GIS severity is greater following WPH ingestion, carbohydrate is likely the preferential macronutrient due to protective mechanisms at the intestinal epithelium, fewer Ex-GIS, and a valuable exogenous fuel source for skeletal muscle (24). Despite this, it is important to acknowledge that some endurance athletes may experience Ex-GIS when consuming higher amounts of carbohydrates (>60–90 g/h carbohydrate) (11) unless they implement a period of gut training (7, 8).

A further consideration when increasing carbohydrate intakes is that athletes may inadvertently increase FODMAP loads due to many carbohydrate foods being associated with a higher FODMAP content, e.g., many grains and cereals are high in fructans. This concept has been shown when a multisport athlete’s habitual diet (moderate in carbohydrate) contained 81 g/d of FODMAP (47), compared to the typical western diet of approximately 25 g/d (44). Therefore, understanding which foods are rich in carbohydrates but lower in FODMAP content is a dietary strategy some athletes who experience Ex-GIS may have to learn how to apply.

It is acknowledged that some athletes used a combination of dietary and non-dietary strategies to manage Ex-GIS and a smaller proportion used non-dietary strategies exclusively. The non-dietary strategies such as medications, relaxation/meditation, or sports psychology were used less frequently than dietary methods to manage Ex-GIS. The use of medications to control Ex-GIS has been reported in research on recreational triathletes, with up to 10% of recreational triathletes regularly experiencing Ex-GIS, reporting the use of medication to manage symptoms (48). Relaxation and psychological interventions (such as cognitive behavior therapy, hypnotherapy, or psychological therapy) are recommended strategies for people with IBS (49). These non-dietary interventions have the potential to reduce Ex-GIS in endurance athletes, but further research is required to confirm efficacy. Observational studies indicate that there may be a link between anxiety or stress and GIS development in athletes (50), but the effect on both functional (e.g., oro-cecal transit time, malabsorption) or integrity changes (e.g., intestinal fatty acid binding protein and cytokine responses) is largely unknown. Further research using randomized controlled cross-over or parallel trials into the use of non-dietary approaches as a strategy to manage Ex-GIS is required, in addition to applying recommended methodological approaches (51).

Given the subtleties of negotiating the most popular dietary strategies for managing Ex-GIS against current sports nutrition guidelines for endurance athletes, it is perhaps not surprising to learn that athletes mainly source support from accredited practicing dietitians. Accredited practicing dietitians are uniquely skilled in addressing health and performance-related dietary requirements. It is suggested that an accredited practicing dietitian experienced in sports nutrition and the administration of acute dietary strategies for managing Ex-GIS be sought (47). Specific nutritional guidance should be provided to individuals who experience Ex-GIS, especially regarding carbohydrate loading and acute carbohydrate intakes before and during exercise. During training, dietary strategies should be trialed to establish individual tolerance and self-perceived efficacy before considering implementation during competition, e.g., a low FODMAP diet or dairy avoidance.

One of the limitations of the current study is that convenience sampling was applied, i.e., athletes were recruited through researcher contacts and specific affiliated social media sites. As all researchers were registered dietitians, it is possible that athletes had a better appreciation of the importance of receiving therapeutic dietary advice through a registered health professional. It is also possible that athletes were from countries with greater access to a registered dietitian, e.g., western vs. non-western countries (47), which may over-represent accredited practicing dietitians as the most common source of nutrition information for managing Ex-GIS.

Endurance athletes try different dietary methods to manage Ex-GIS, e.g., dietary fiber reduction, a low FODMAP diet, dairy avoidance, or increasing carbohydrates. All of these dietary strategies tried were rated as successful. Further research is required to better understand the most appropriate dietary strategies for endurance athletes with Ex-GIS and if these are best advocated acutely before exercise or integrated into an athlete’s habitual diet. Gaining an appreciation of athletes’ self-selected nutrition strategies in managing Ex-GIS may enable further refinement of strategies recommended to manage Ex-GIS. This is especially applicable for accredited practicing dietitians whom athletes often seek for nutrition information to manage Ex-GIS.

## Data availability statement

The raw data supporting the conclusions of this article will be made available by the authors, without undue reservation.

## Ethics statement

The studies involving human participants were reviewed and approved by the Human Research Ethics Committee—University of the Sunshine Coast, Australia (ethics approval number: S201402). The patients/participants provided their written informed consent to participate in this study.

## Author contributions

RS and GS: conceptualization and writing—original draft. RS, GS, RC, FP, and DL: methodology and writing—review and editing. RS: statistical analysis and project administration. GS: supervision. All authors contributed to the article and approved the submitted version.
