# Supplementary material for: An exploratory study of the management strategies reported by endurance athletes with exercise-associated gastrointestinal symptoms
Source: Front Nutr. 2022 Nov 9;9:1003445. doi: 10.3389/fnut.2022.1003445 (PMC9691682; doi:10.3389/fnut.2022.1003445)
Supplement: Supplementary file 1 [file Data_Sheet_1.PDF]

Endurance Athletes Questionnaire Final

(Please note: This questionnaire is designed for online purposes, drop-down boxes unavailable)

Start of Block: Introduction

Introduction

QUESTIONNAIRE

Endurance Athletes' Sports Nutrition Beliefs, Knowledge, Information Sources, and Intended Practices to Prevent or Manage Gastrointestinal Symptoms Around Exercise

Thank you for taking the time to complete this questionnaire. By completing this questionnaire, you will help us to better understand endurance athletes' sports nutrition beliefs, knowledge, information sources, and intended practices for training and events and how to prevent or manage gastrointestinal (gut) symptoms around exercise. Endurance sport is defined as a continuous aerobic activity for longer than 60 minutes. If you are 18 years or over and you train or compete in events longer than 60 minutes, you are invited to complete the questionnaire. The questionnaire will take about 10 - 15 minutes of your time.

INTRODUCTION

Please read the Research Participation Information Sheet (Please READ) and confirm your consent to participate below.

○ Yes, I provide consent

○ No, I do not provide consent

Skip To: End of Survey If QUESTIONNAIRE Endurance Athletes' Sports Nutrition Beliefs, Knowledge, Information Sources, and... = No, I do not provide consent

---

Sign **Yes, I provide consent to participate in this research project (please sign below)**

Code **For coding purposes, please provide a participant code i.e. First 3 letters of your name and last 3 numbers of your phone number**

____

End of Block: Introduction

Start of Block: PART A DEMOGRAPHICS

PART A
DEMOGRAPHICS
The information collected in this section provides data regarding research participants, necessary to describe the survey sample and overall responses. Please complete this questionnaire based on the main endurance event (e.g., Ironman triathlon), you train or compete in currently and your experiences over the last 12 months.

Q1 **What is your biological sex?**

- ☐ Male
- ☐ Female
- ☐ Prefer to not disclose
- ☐ Prefer to self-describe ____

Q2 **What is your age in years?**
Years

▼ 18 ... 117

Q3 **Select the main endurance sport or event you train or compete in currently. Please specify the distance or type of event in the text box? For example, Triathlon, Olympic distance**

N.B. Please answer the remaining questions based on this event.

- ○ Triathlon ____
- ○ Cycling ____
- ○ Running ____
- ○ Swimming ____
- ○ Walking ____
- ○ Multi-sport ____
- ○ Other (Please specify) ____

Q4 **Specify if the event you compete in is a single day or multi-day event, and how long it usually takes you to complete? Please select both hours and minutes.**
Single day or Multi-day event
Hours
Minutes

▼ Single day event ... Multi-Day event - Greater than 5 day ~ Greater than 12 hour daily average ~ 0

Q5 **How would you best describe yourself and your level of training?**

▼ Recreational Non-competitive ... Competitive International i.e., Compete at World, Commonwealth or Olympic events

---

Q6 **During your heaviest training week, how many hours do you typically spend on aerobic endurance training for your sport or event?**
TOTAL hours per week

▼ 1 ... 50+

End of Block: PART A DEMOGRAPHICS

Start of Block: PART B CARBOHYDRATE KNOWLEDGE

PART B
CARBOHYDRATE KNOWLEDGE

The following questions are to determine your current knowledge about carbohydrate and where you obtain your sports nutrition knowledge.

Q7 **From the following list, select the food and drinks you recognise as carbohydrate or non-carbohydrate choices? (Please note: A response is required for each item)**

	Carbohydrate choices	Non-carbohydrate choices
100 g grilled chicken	○	○
2 slices of wholemeal bread	○	○
1 cup of raw rolled oats	○	○
1 medium baked potato	○	○
100 g baked salmon	○	○
1 cup cooked pasta	○	○
1 cup cooked white rice	○	○
4 large leaves of iceberg lettuce	○	○
300 mL of sports drink (e.g., Gatorade or Powerade)	○	○
1 large banana	○	○
300 mL soft drink/ soda (e.g., lemonade or cola)	○	○
2 wheat biscuits (e.g., Weetbix)	○	○
1/2 a large avocado	○	○
1 cup raw broccoli	○	○
2 boiled eggs	○	○

Q8 **From the drop-down list below, select the MOST important information source that has influenced your nutrition practices in relation to your sport.**
Please note: The following list is currently in alphabetical order.

▼ I have never considered nutrition in my sport ... Other, please specify

End of Block: PART B CARBOHYDRATE KNOWLEDGE

Start of Block: PART C CARBOHYDRATE BELIEFS

PART C
CARBOHYDRATE BELIEFS

The following questions are in relation to your carbohydrate beliefs and the effects it may (or may not) have on exercise performance.

Q10 **From the drop-down boxes, select how much you agree or disagree with the following statements about endurance athletes consuming additional carbohydrates around training sessions or events to improve athletic performance.**
Compared to a non-training day or rest day, endurance athletes would benefit from increasing their carbohydrate intake...

	Please select one answer
1 – 2 days **BEFORE** an event 90 minutes (1.5 hours) or longer	▼ Strongly Disagree ... Strongly Agree
In the last meal or snack **BEFORE** endurance training or an event 90 minutes (1.5 hours) or longer	▼ Strongly Disagree ... Strongly Agree
**DURING** endurance training or an event (e.g., sports drinks or gels) lasting 60 minutes (1 hour) or longer	▼ Strongly Disagree ... Strongly Agree
Within 30 minutes **AFTER** endurance exercise during recovery when there are less than 8 hours between two fuel demanding sessions	▼ Strongly Disagree ... Strongly Agree

End of Block: PART C CARBOHYDRATE BELIEFS

Start of Block: PART D INTENDED PRACTICES

PART D
INTENDED NUTRITION PRACTICES

The following questions are based on what you plan to do with your food intake, energy (calories/ kilojoules), and macronutrients (carbohydrate, protein, and fat) before, during, or after an endurance training day or an event. Please consider the main sport or event you are currently training or competing in.

Q10 **Do you implement any specific nutrition strategies (i.e., change your usual eating habits or change the types of foods and drinks you usually consume) before, during or after endurance training or an event?**

○ Yes (Please specify) ____

○ No

Skip To: End of Block If Do you implement any specific nutrition strategies (i.e., change your usual eating habits or change... = No

---

Q11 **Please consider what you plan to do for each scenario (in your main sport or event).**

Select from the drop-down boxes, whether you plan to eat LESS, the SAME, or MORE energy (calories/ kilojoules), carbohydrate, protein, or fat, compared to a non-training or lighter training day?
N.B. This includes both solid and liquid foods.

	Energy (calories/ kilojoules)	Carbohydrate	Protein	Fat
1 – 2 days **BEFORE** an event 90 minutes (1.5 hours) or longer	▼ Plan to eat LESS ... Plan to eat MORE	▼ Plan to eat LESS ... Plan to eat MORE	▼ Plan to eat LESS ... Plan to eat MORE	▼ Plan to eat LESS ... Plan to eat MORE
In the last meal or snack **BEFORE** endurance training or an event 90 minutes (1.5 hours) or longer	▼ Plan to eat LESS ... Plan to eat MORE	▼ Plan to eat LESS ... Plan to eat MORE	▼ Plan to eat LESS ... Plan to eat MORE	▼ Plan to eat LESS ... Plan to eat MORE
**DURING** endurance training or an event (i.e., sports drinks or gels) lasting 60 minutes (1 hour) or longer	▼ Plan to eat LESS ... Plan to eat MORE	▼ Plan to eat LESS ... Plan to eat MORE	▼ Plan to eat LESS ... Plan to eat MORE	▼ Plan to eat LESS ... Plan to eat MORE
Within 30 minutes **AFTER** endurance exercise during recovery when there are less than 8 hours between two fuel demanding sessions	▼ Plan to eat LESS ... Plan to eat MORE	▼ Plan to eat LESS ... Plan to eat MORE	▼ Plan to eat LESS ... Plan to eat MORE	▼ Plan to eat LESS ... Plan to eat MORE

End of Block: PART D INTENDED PRACTICES

Start of Block: PART E EIGS

PART E
EXERCISE ASSOCIATED GASTROINTESTINAL SYMPTOMS

Before, during, or after endurance exercise some athletes experience gastrointestinal symptoms e.g., belching, nausea, bloating, diarrhoea, etc. The following questions relate to gastrointestinal

symptoms, and symptom severity experienced before, during, or after training and/ or competition and information sources.

Q12 **Do you experience** gastrointestinal symptoms **(e.g., belching, nausea, bloating, diarrhoea etc.) before, during or after endurance training and/ or competition?**

○ Yes

○ No

Skip To: End of Block If Do you experience gastrointestinal symptoms (e.g., belching, nausea, bloating, diarrhoea etc.) bef... = No

---

Q13 **When do you experience gastrointestinal symptoms most frequently?**

○ Around (before, during and/or after) Training

○ Around (before, during and/or after) Competitions

○ Equally around training and competitions

Skip To: Q23 If When do you experience gastrointestinal symptoms most frequently? = Around (before, during and/or after) Competitions

---

Q14 **Do you experience** gastrointestinal symptoms ***BEFORE*** **training?**

○ Yes

○ No

Skip To: Q17 If Do you experience gastrointestinal symptoms BEFORE training? = No

---

Q15 **When do your gastrointestinal symptoms start BEFORE training?**
Please select the most common time that your symptoms start.
Before exercise (select time)

▼ No consistent time before exercise starts ... 1 hour before exercise starts

---

Q16 **From the drop-down list below please select the severity of the different gastrointestinal symptoms you experience *BEFORE* training. (Note: NO response equals NO symptoms)**

No symptoms = 0; Mild symptoms = 1 – 4 (i.e., sensation of GIS, but not substantial enough to interfere with exercise workload); Severe symptoms = 5 – 9 (i.e., GIS substantial enough to interfere with exercise workload); Extremely severe symptoms = 10 (i.e. indicative of extreme GIS warranting exercise cessation). Adapted from Modified Visual Analogue Scale, Gaskell, et al. (2019) IJSNEM.

	Symptom Severity
Belching	▼ 0 = No Symptoms ... 10 = Extremely Severe Symptoms
Heartburn	▼ 0 = No Symptoms ... 10 = Extremely Severe Symptoms
Bloating (Stomach fullness)	▼ 0 = No Symptoms ... 10 = Extremely Severe Symptoms
Stomach pain	▼ 0 = No Symptoms ... 10 = Extremely Severe Symptoms
Urge to regurgitate	▼ 0 = No Symptoms ... 10 = Extremely Severe Symptoms
Regurgitation	▼ 0 = No Symptoms ... 10 = Extremely Severe Symptoms
Projectile vomiting (Great than 0 = Vomiting)	▼ 0 = No Symptoms ... 10 = Extremely Severe Symptoms
Flatulence	▼ 0 = No Symptoms ... 10 = Extremely Severe Symptoms
Lower abdominal bloating (Abdominal pressure)	▼ 0 = No Symptoms ... 10 = Extremely Severe Symptoms
Urge to defecate	▼ 0 = No Symptoms ... 10 = Extremely Severe Symptoms
Left intestinal pain	▼ 0 = No Symptoms ... 10 = Extremely Severe Symptoms
Defecation: Loose stools (Greater than 0 = Loose stools)	▼ 0 = No Symptoms ... 10 = Extremely Severe Symptoms
Defecation: Diarrhoea (Greater than 0 = Diarrhoea)	▼ 0 = No Symptoms ... 10 = Extremely Severe Symptoms
Defecation: Bloody Stools (Greater than 0 = Bloody stools)	▼ 0 = No Symptoms ... 10 = Extremely Severe Symptoms
Nausea	▼ 0 = No Symptoms ... 10 = Extremely Severe Symptoms
Dizziness	▼ 0 = No Symptoms ... 10 = Extremely Severe Symptoms
Stitch (Acute transient abdominal pain)	▼ 0 = No Symptoms ... 10 = Extremely Severe Symptoms

Q17 **Do you experience** gastrointestinal symptoms ***DURING*** **training?**

◯ Yes

◯ No

Skip To: Q20 If Do you experience gastrointestinal symptoms DURING training? = No

Q18 ***DURING*** **training when do your gastrointestinal symptoms usually start?**
Please select the most common time your symptoms start.
During exercise (select time)

▼ Residual effects from symptoms experienced before exercise ... More than 24 hours during exercise

Q19 **From the drop-down list below please select the severity of the different gastrointestinal symptoms you experience *DURING* training.**
(Note: NO response equals NO symptoms)

(Same chart as Q16)

Q20 **Do you experience** gastrointestinal symptoms ***AFTER*** **training?**

◯ Yes

◯ No

Skip To: Q23 If Do you experience gastrointestinal symptoms AFTER training? = No

Q21 ***AFTER*** **training when do your gastrointestinal symptoms usually start?**
Please select the most common time your symptoms start.
After exercise (select time)

▼ Residual effects from symptoms experienced during exercise ... More than 12 hours after exercise

Q22 **From the drop-down list below please select the severity of the different gastrointestinal symptoms you experience *AFTER* training. (Note: NO response equals NO symptoms)**

(Same chart as Q16)

Q23 **Do you experience** gastrointestinal symptoms ***BEFORE*** **competitions?**

○ Yes

○ No

Skip To: Q26 If Do you experience gastrointestinal symptoms BEFORE competitions? = No

Q24 ***BEFORE*** **competitions when does your gastrointestinal symptoms usually start?**
Please select the most common time your symptoms start.
Before competitions (Select time)

▼ No consistent time before exercise starts ... 1 hour before exercise starts

Q25 **From the drop-down list below please select the severity of the different gastrointestinal symptoms you experience *BEFORE* competitions. (Note: NO response equals NO symptoms)**

(Same chart as Q16)

Q26 **Do you experience** gastrointestinal symptoms *DURING* **competitions?**

○ Yes

○ No

Skip To: Q29 If Do you experience gastrointestinal symptoms DURING competitions? = No

Q27 ***DURING* competitions when does your gastrointestinal symptoms usually start?**
Please select the most common time your symptoms start.
During competitions (Select time)

▼ Residual effects from symptoms experienced before exercise ... More than 24 hours during exercise

Q28 **From the drop-down list below please select the severity of the different gastrointestinal symptoms you experience *DURING* competitions.**
(Note: NO response equals NO symptoms)

(Same chart as Q16)

Q29 **Do you experience gastrointestinal symptoms *AFTER* competitions?**

- ○ Yes
- ○ No

Skip To: Q32 If Do you experience gastrointestinal symptoms AFTER competitions? = No

Q30 ***AFTER* competitions when do your gastrointestinal symptoms usually start?**
Please select the most common time your symptoms start.
After competitions (Select time)

▼ Residual effects from symptoms experienced during exercise ... More than 12 hours after exercise

Q31 **From the drop-down list below please select the severity of the different gastrointestinal symptoms you experience *AFTER* competitions.**
(Note: NO response equals NO symptoms)

(Same chart as Q16)

Q32 **From the drop-down list below, select the MOST important information source that has influenced your nutrition practices regarding the management of your gastrointestinal symptoms. Please note: The following list is currently in alphabetical order.**

▼ I have never sought information to reduce symptoms ... Other, please specify

End of Block: PART E EIGS

Start of Block: PART F INTENDED NUTRITION PRACTICE FOR GIS

PART F
INTENDED NUTRITION PRACTICES FOR GASTROINTESTINAL SYMPTOMS

The following questions relate to dietary or non-dietary strategies that you have tried to reduce gastrointestinal symptoms before, during or after exercise.

Q33 **Have you tried any strategies (dietary or non-dietary) to reduce the development of gastrointestinal symptoms before, during or after exercise.**

○ Yes (Please specify, before, during or after and what you have tried) ____

○ No

Skip To: End of Block If Have you tried any strategies (dietary or non-dietary) to reduce the development of gastrointestinal... = No

---

Q34 **Please select all the dietary components you have tried eating more or less of to reduce the development of your gastrointestinal symptoms (GIS).**
NB: Please select "Not tried" if you have not tried changing that dietary component.

	BEFORE exercise (Last meal or snack before exercise)	DURING Exercise	AFTER exercise (Within 30 minutes of exercise completion)	Dietary change made, but not specifically related to exercise	Success of dietary component in reducing GIS	Not tried to change this dietary component
						Not Tried
Carbohydrate	▼ More ... Less	▼ More ... Less	▼ More ... Less	▼ More ... Less	▼ Significantly Worse ... Significant Improvement	☐
Protein	▼ More ... Less	▼ More ... Less	▼ More ... Less	▼ More ... Less	▼ Significantly Worse ... Significant Improvement	☐
Fat	▼ More ... Less	▼ More ... Less	▼ More ... Less	▼ More ... Less	▼ Significantly Worse ... Significant Improvement	☐
Fibre	▼ More ... Less	▼ More ... Less	▼ More ... Less	▼ More ... Less	▼ Significantly Worse ... Significant Improvement	☐
Coffee or caffeine	▼ More ... Less	▼ More ... Less	▼ More ... Less	▼ More ... Less	▼ Significantly Worse ... Significant Improvement	☐
Water or fluid	▼ More ... Less	▼ More ... Less	▼ More ... Less	▼ More ... Less	▼ Significantly Worse ... Significant Improvement	☐

Q35 **Please review the table and select all the dietary strategies you have tried to reduce your gastrointestinal symptoms.**
NB: Please select "Not tried" if you have not tried that dietary strategy.
***FODMAPs (Fermentable Oligosaccharides Disaccharides, Monosaccharides, and Polyols)**

	**BEFORE** exercise (Last meal or snack before exercise)	**DURING** Exercise	**AFTER** exercise (Within 30 minutes of exercise completion)	Dietary change made, but not specifically related to exercise	Success of dietary component in reducing GIS	Not tried to change this dietary component
	Tried	Tried	Tried	Tried		Not Tried
Gluten-Free	▢	▢	▢	▢	▼ Significantly Worse ... Significant Improvement	▢
Wheat-Free	▢	▢	▢	▢	▼ Significantly Worse ... Significant Improvement	▢
Lactose-Free	▢	▢	▢	▢	▼ Significantly Worse ... Significant Improvement	▢
Dairy-Free	▢	▢	▢	▢	▼ Significantly Worse ... Significant Improvement	▢
Low FODMAPs*	▢	▢	▢	▢	▼ Significantly Worse ... Significant Improvement	▢

Q36 **Have you tried any other specific dietary strategies to reduce gastrointestinal symptoms?**

○ Yes (Please specify) ____

○ No

Skip To: Q37 If Have you tried any other specific dietary strategies to reduce gastrointestinal symptoms? = No

Q37 **Please review the table and select all the dietary supplements you have tried to reduce your gastrointestinal symptoms.**
NB: Please select "Not tried" if you have not tried that dietary supplement.

	**BEFORE** exercise (Last meal or snack before exercise)	**DURING** Exercise	**AFTER** exercise (Within 30 minutes of exercise completion)	**TRIED** this dietary supplement but not specifically related to exercise	**NOT TRIED** this dietary supplement	Success of dietary supplements in reducing GIS
	Tried	Tried	Tried	Tried	Not tried	
Glutamine	☐	☐	☐	☐	☐	▼ Significantly Worse ... Significantly Improvement
L-Citrulline	☐	☐	☐	☐	☐	▼ Significantly Worse ... Significantly Improvement
Arginine	☐	☐	☐	☐	☐	▼ Significantly Worse ... Significantly Improvement
Bovine Colostrum	☐	☐	☐	☐	☐	▼ Significantly Worse ... Significantly Improvement
Curcumin	☐	☐	☐	☐	☐	▼ Significantly Worse ... Significantly Improvement
Probiotics	☐	☐	☐	☐	☐	▼ Significantly Worse ... Significantly Improvement
Prebiotics	☐	☐	☐	☐	☐	▼ Significantly Worse ... Significantly Improvement
Synbiotics	☐	☐	☐	☐	☐	▼ Significantly Worse ... Significantly Improvement
Antioxidants (e.g., Vitamin C or E)	☐	☐	☐	☐	☐	▼ Significantly Worse ... Significantly Improvement
Nitrates (e.g., Beetroot juice)	☐	☐	☐	☐	☐	▼ Significantly Worse ... Significantly Improvement

Q38 **Have you tried any other dietary supplements to reduce your gastrointestinal symptoms?**

○ Yes (Please list type of dietary supplement and when they are consumed) ____

○ No

Q39 **Please select all of the other strategies you have tried to reduce your gastrointestinal symptoms, and any comments regarding this strategy i.e., timing, dose.**

▢ Medications (please list) ____

▢ Relaxation/ meditation ____

▢ Herbal preparations ____

▢ Acupuncture ____

▢ Sports Psychology ____

▢ Portion size manipulation ____

▢ All liquid diet pre-event ____

▢ Other strategy (please specify) ____

Q40 **From the drop-box boxes select the MOST successful dietary or non-dietary strategy that you have tried to reduce your gastrointestinal symptoms.**
Dietary or Non-Dietary strategy
Specific Strategy
Specific Timing
Select more or less if applicable

▼ Dietary Components e.g., Carbohydrate ... Other strategies e.g., Medications ~ Other specified above ~ ~

Q41 If not listed above, please list the dietary or non-dietary strategies that has been the most successful to reduce your gastrointestinal symptoms around exercise.

____

End of Block: PART F INTENDED NUTRITION PRACTICE FOR GIS

Start of Block: PART G DISEASES OR DISORDERS OF THE GI TRACT

PART G **PART G**
DISEASES OR DISORDERS OF THE GASTROINTESTINAL TRACT

The following questions are based on diseases or disorders of the gastrointestinal tract, and various treatment methods you may/ may not have tried.

Q42 **Do you suffer from a diagnosed gastrointestinal disease or disorder?**

○ Yes

○ No

Skip To: End of Block If Do you suffer from a diagnosed gastrointestinal disease or disorder? = No

Q43 **Please select your diagnosed gastrointestinal disease or disorder.**
Specific Disease or Disorder Group
Specific Disease or Disorder Name
Specific Disease or Disorder name (defined)

▼ A. Inflammatory Bowel Disorders ... Unsure of Diagnosis ~ Unsure of Diagnosis ~ Unsure of Diagnosis

Q44 Please list the dietary or non-dietary strategies that has been the most successful to reduce your gastrointestinal symptoms.

____

End of Block: PART G DISEASES OR DISORDERS OF THE GI TRACT
